# Listeriomics: an Interactive Web Platform for Systems Biology of *Listeria*

**DOI:** 10.1128/mSystems.00186-16

**Published:** 2017-03-14

**Authors:** Christophe Bécavin, Mikael Koutero, Nicolas Tchitchek, Franck Cerutti, Pierre Lechat, Nicolas Maillet, Claire Hoede, Hélène Chiapello, Christine Gaspin, Pascale Cossart

**Affiliations:** a Département de Biologie Cellulaire et Infection, Institut Pasteur, Unité des Interactions Bactéries-Cellules, Paris, France; b INSERM, U604, Paris, France; c INRA, USC2020, Paris, France; d Institut Pasteur–Bioinformatics and Biostatistics Hub, C3BI, USR 3756 IP CNRS, Paris, France; e CEA-Division of Immuno-Virology, Institute of Emerging Diseases and Innovative Therapies, IDMIT Center, Fontenay-aux-Roses, France; f INRA-UR MIAT, Université de Toulouse, Castanet-Tolosan, France; NYU School of Medicine

**Keywords:** *Listeria*, transcriptomics, database, genomics, proteomics, systems biology

## Abstract

As for many model organisms, the amount of *Listeria* omics data produced has recently increased exponentially. There are now >80 published complete *Listeria* genomes, around 350 different transcriptomic data sets, and 25 proteomic data sets available. The analysis of these data sets through a systems biology approach and the generation of tools for biologists to browse these various data are a challenge for bioinformaticians. We have developed a web-based platform, named Listeriomics, that integrates different tools for omics data analyses, i.e., (i) an interactive genome viewer to display gene expression arrays, tiling arrays, and sequencing data sets along with proteomics and genomics data sets; (ii) an expression and protein atlas that connects every gene, small RNA, antisense RNA, or protein with the most relevant omics data; (iii) a specific tool for exploring protein conservation through the *Listeria* phylogenomic tree; and (iv) a coexpression network tool for the discovery of potential new regulations. Our platform integrates all the complete *Listeria* species genomes, transcriptomes, and proteomes published to date. This website allows navigation among all these data sets with enriched metadata in a user-friendly format and can be used as a central database for systems biology analysis.

**IMPORTANCE** In the last decades, *Listeria* has become a key model organism for the study of host-pathogen interactions, noncoding RNA regulation, and bacterial adaptation to stress. To study these mechanisms, several genomics, transcriptomics, and proteomics data sets have been produced. We have developed Listeriomics, an interactive web platform to browse and correlate these heterogeneous sources of information. Our website will allow listeriologists and microbiologists to decipher key regulation mechanism by using a systems biology approach.

## Abstract (precis)

In the last decades, *Listeria* has become a key model organism for the study of host-pathogen interactions, noncoding RNA regulation, and bacterial adaptation to stress. To study these mechanisms, several genomics, transcriptomics, and proteomics data sets have been produced. We have developed Listeriomics, an interactive web platform to browse and correlate these heterogeneous sources of information. Our website will allow listeriologists and microbiologists to decipher key regulation mechanism by using a systems biology approach.

## INTRODUCTION

*Listeria monocytogenes* is a foodborne pathogen responsible for foodborne infections with a mortality rate of 25%. This pathogen is responsible for gastroenteritis, sepsis, and meningitis and can cross three host barriers, the intestinal, placental, and blood-brain barriers. It is a major concern for pregnant women, as it induces abortions (1). *L. monocytogenes* can enter, replicate in, and survive in a wide range of human cell types, such as macrophages, epithelial cells, and endothelial cells. Moreover, *Listeria* has emerged as a model organism for the study of host-pathogen interactions (1–3).

*Listeria* belongs to the *Firmicutes* phylum. The *Listeria* genus is made up of the widely studied pathogenic species *L. monocytogenes*; another pathogenic species, *Listeria ivanovii*, that mostly affects ruminants; and 15 nonpathogenic species (4–10). In 2001, the genomes of *L. monocytogenes* strain EGD-e and one *Listeria innocua* strain were sequenced (11). Since then, many other *Listeria* genomes, covering all the lineages, have been sequenced (12–17). Currently, the NCBI refSeq database contains 83 complete *Listeria* genomes, including 70 *L. monocytogenes* genomes. The number of *Listeria* strains sequenced will probably grow exponentially in the coming years. Efforts have been made to summarize all these genomes on specific databases like ListiList (11), GenoList (18), GECO-LisDB server (16), and ListeriaBase (19) to find common gene features and to develop pangenome studies of *Listeria* species.

The first *Listeria* transcriptomic data set was published in 2007 (20). Since that report, 64 ArrayExpress studies, corresponding to 362 different biological conditions, have been produced (21). Only seven of them are transcriptome sequencing (RNA-Seq) studies, and all the others correspond to transcription profiling by microarrays, with the EGD-e strain being the most frequently used strain. *Listeria* is also a key organism in the study of bacterial regulatory small noncoding RNAs (sRNAs). Despite the high number of studies on *Listeria* noncoding RNAs, only two websites with *Listeria*-related data sets have been published. The first one is a genome viewer published along with a transcription start site (TSS) study of *Listeria* (22). The second is the sRNAdb database (23), which provides tools to visualize the conservation of gene loci surrounding noncoding RNAs in different Gram-positive bacteria.

The ability of *L. monocytogenes* to enter into various types of cells is due to the variety of proteins it secretes or anchors to its cell wall and external membrane. Consequently, many proteomic studies have been performed to analyze the exoproteome of *Listeria* (24–35). Other studies have focused on cytoplasmic proteins (27, 33, 35–44). To our knowledge, 74 proteome studies have been conducted to decipher the production and localization of *Listeria* proteins. Nevertheless, no database exists that combines all these proteomics data sets into a single, user-friendly resource.

The number of omics data sets produced has increased exponentially. The number of tools to analyze these data, as well as the diversity of databases to store them, has also burgeoned. In parallel with this increase, many efforts have been made to develop accurate web-based tools to integrate diverse omics data for each model organism. One of the most complete resources is certainly the University of California Santa Cruz Encyclopedia of DNA Elements (ENCODE at UCSC) Genome Browser (45), which allows the visualization of a large variety of human and mouse omics data sets. For prokaryotic organisms, the BioCyc (46, 47) and Pathosystems Resource Integration Center (48) websites have been created. These websites connect all the published genomic and transcriptomic data sets for prokaryotic organisms to metabolic pathways. Such wide-ranging web resources are useful for microbiologists, but for in-depth analyses, the development of individual web resources with curated metadata per model organism is also required. In the case of bacteria, few heterogeneous omics data sets are available (49) and few model organisms have dedicated web resources, including *Escherichia coli*, with RegulonDB (50) and PortEco (51), and *Bacillus subtilis*, with SubtiWiki (52). As yet, resources for *Listeria* species are limited.

Here, we present Listeriomics (http://listeriomics.pasteur.fr/), a highly interactive web resource summarizing many omics data sets related to the genus *Listeria*. We have curated and integrated all the available *Listeria* transcriptomic, proteomic, and genomic data sets to date. The Listeriomics platform was developed not only to integrate these diverse data sets but also to display them in a single viewer. To interactively explore these data sets, our website also provides different tools, i.e., (i) a genome viewer for displaying gene expression arrays, tiling arrays, and sequencing data, along with proteomic and genomic data sets; (ii) an expression atlas and protein atlas, inspired by the EBI Expression Atlas, that connects genomic elements (genes, small RNAs, antisense RNAs [asRNAs]) to the most relevant omics data; (iii) a specific tool for exploring protein conservation through the *Listeria* phylogenomic tree; and (iv) a coexpression network analysis tool for the discovery of potential new regulations.

## RESULTS

### The Listeriomics web interface.

**FIG 1:**
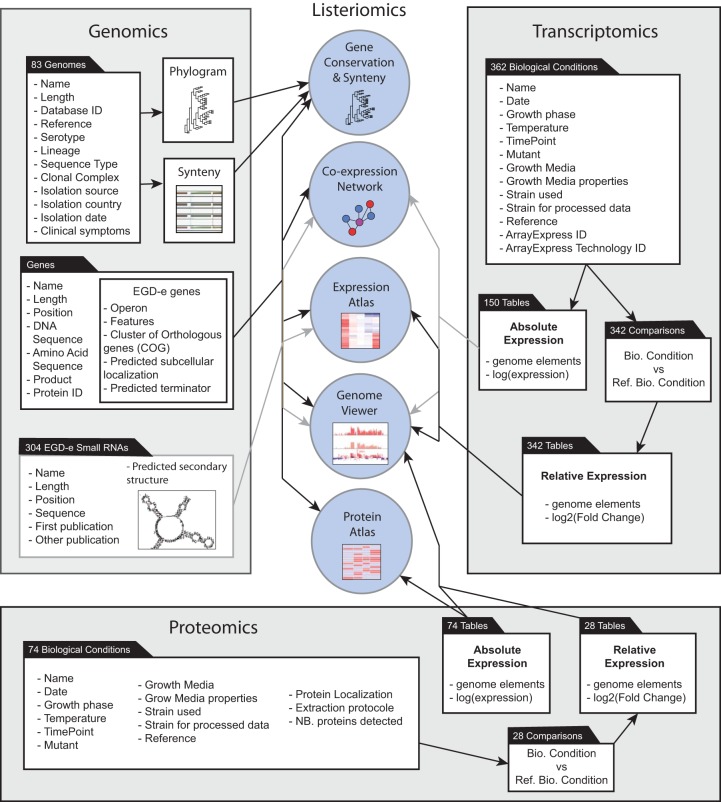
Overview of the Listeriomics platform. (Center) The five major tools of Listeriomics, i.e., gene conservation and synteny, coexpression network, genome viewer, expression, and protein atlas. (Left) Summary of all the available genomic information available on the website. (Right) List of all the transcriptomic information available in Listeriomics. (Bottom) View of all the proteomic information that can be accessed.

Genomic, transcriptomic, or proteomic data can be browsed by using the Listeriomics website (http://listeriomics.pasteur.fr/) main page (Fig. 1; Table 1; see Fig. S1 in the supplemental material). For each type of data, we designed a summary panel to navigate through the different data sets. The top banner of the website gives direct access to them. As summarized in Table 1, users can search 83 complete *Listeria* genomes and browse 492 transcriptome and 74 proteome data sets. Listeriomics integrates four tools for omics data management, i.e., (i) a genome viewer for displaying gene expression array, tiling array, and sequencing data along with proteomics and genomics data; (ii) an expression atlas and protein atlas that connect every genomic element (genes, small RNAs, asRNAs) to the most relevant omics data; (iii) a protein conservation tool for the direct visualization of the presence or absence of a protein in a specific *Listeria* strain; and (iv) a coexpression network analysis tool for the visualization of genome features with the same expression profile.

10.1128/mSystems.00186-16.1 FIG S1  Flowchart of omics data set integration in the Listeriomics database. (A) Complete genome sequences from the RefSeq and GenBank databases were downloaded and integrated into Listeriomics, along with pathway information and small RNAs. (B) MAGE-TAB data sets were downloaded from ArrayExpress. Metadata on the data sets were manually curated, and processed gene expression array tables were added. Raw RNA-Seq data were downloaded and mapped to a reference genome. After log fold change calculation, all of the data sets were normalized with variance normalization to fix the statistical deviation at 1 and ensure comparability. (C) Proteomics data sets were manually curated from the core articles and related supplementary data. Download FIG S1, EPS file, 1.1 MB. Copyright © 2017 Bécavin et al. 2017 Bécavin et al. This content is distributed under the terms of the Creative Commons Attribution 4.0 International license.

**TABLE 1:** Summary of omics data sets included in the Listeriomics database

Category	Data sizes	Data type(s)	Tools available
Genomics	83 complete genomes (NCBI), all protein coding genes and noncoding RNAs, 304 small RNAs	Genome, phylogeny, genome elements, homologs	Genome summary, gene panel, small RNA panel, genome viewer
Transcriptomics	362 biological conditions, 8 *Listeria* strains, 342 comparisons	Gene expression array, tiling array, TSS, RNA-Seq	Transcriptome summary, expression atlas, heat map, genome viewer
Proteomics	74 biological conditions, 4 *Listeria* strains, 28 comparisons	Mass spectrometry	Proteome summary, protein atlas, heat map, genome viewer

The genomic interface is designed to browse every complete genome of the Listeriomics resource. Users can access strain name, serotype, lineage, and isolation information, along with a complete phylogenomic tree of *Listeria* strains (Fig. 1). From this table, scientists can access all the annotated genes of a specific strain. For each *Listeria* gene, five different information panels are available. The first panel shows all the general information about the position of the gene, its predicted annotated function. DNA and amino acid sequences can be accessed and saved as FASTA files or sent directly for a BLASTn or BLASTp search (53). The predicted subcellular localization (cytoplasm, cytoplasmic membrane, cell wall, cell surface, and extracellular milieu [27]) of each protein is also displayed along with information about the secretion pathway possibly used by the protein. The second panel provides an instant view of the conservation of a specific protein in other *Listeria* strains. This panel dynamically displays homologs on the *Listeria* reference tree in each existing *Listeria* strain. It also displays a summary table of all the homologous proteins with their similarity percentages and amino acid sequences. Users can also create a multialignment file of the homologous proteins. With the third panel, the user can visualize the protein locus synteny in all *Listeria* strains. We built an external synteny website by using the SynTView architecture (54). A fourth panel uses the expression atlas to show in which transcriptomics data sets the selected gene is differently expressed. The fifth panel displays every proteomics data set in which the protein encoded by the selected gene has been detected. Finally, from the home webpage, a summary panel with all the small RNAs in *L. monocytogenes* EGD-e can be accessed (Fig. 1). For each noncoding RNA element, one can display its position, its nucleotide sequence, its predicted secondary structure at 37°C, and a table displaying all supplementary information provided in source references (22, 55–58).

**FIG 2:**
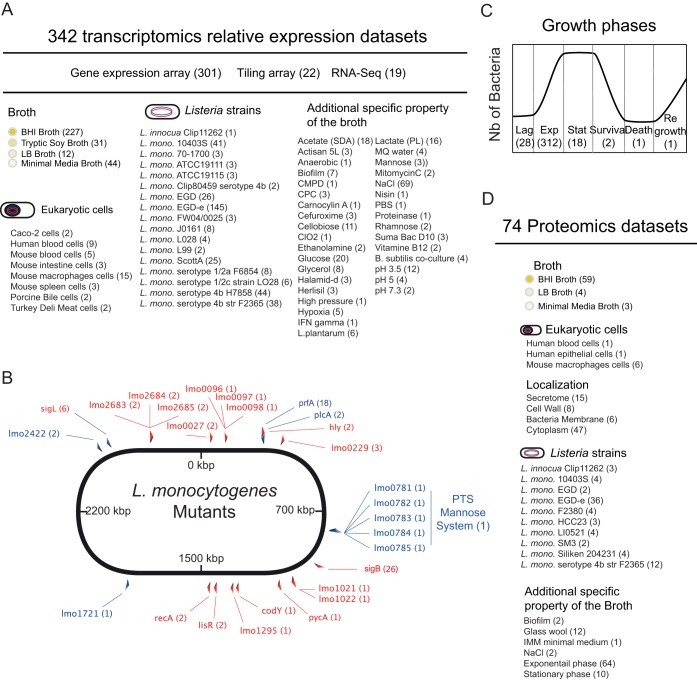
Transcriptomic and proteomic data sets available in the Listeriomics database. (A) Summary of all the transcriptomic data sets available at the Listeriomics website. In parentheses is the number of transcriptomics data sets available in the Listeriomics database for a specific biological condition. (B) Schematic representation of all the *L. monocytogenes* mutants for which transcriptomic data sets are available in the Listeriomics database. (C) Schematic representation of the number of transcriptomics data sets available for each *L. monocytogenes* growth phase. (D) Summary of all the proteomics data sets available at the Listeriomics website. In parentheses is the number of proteomics data sets available in the Listeriomics database for a specific biological condition.

In the transcriptomic interface of the Listeriomics website, researchers can access all the *Listeria* transcriptomic data sets published so far. A searchable table shows every data set available with a precise description of the biological conditions studied. In total, four different transcriptome technologies (gene expression array, tiling array, RNA-Seq, and TSS) are included for seven different *L. monocytogenes* strains grown in four different broth media under seven intracellular conditions (Fig. 2A). Once transcriptomic data sets have been selected, it is possible to obtain the number of genes and small RNAs that are differently expressed between selected data sets and their corresponding reference biological conditions. Users can then directly visualize the relative gene expression values, shown as log fold changes, in a heat map representation. Also, specific lists of elements extracted from key publications can be chosen, such as a list of internalin genes (17) or surface proteins with the LPXTG motif (59), to display their expressions under specific biological conditions.

**FIG 3:**
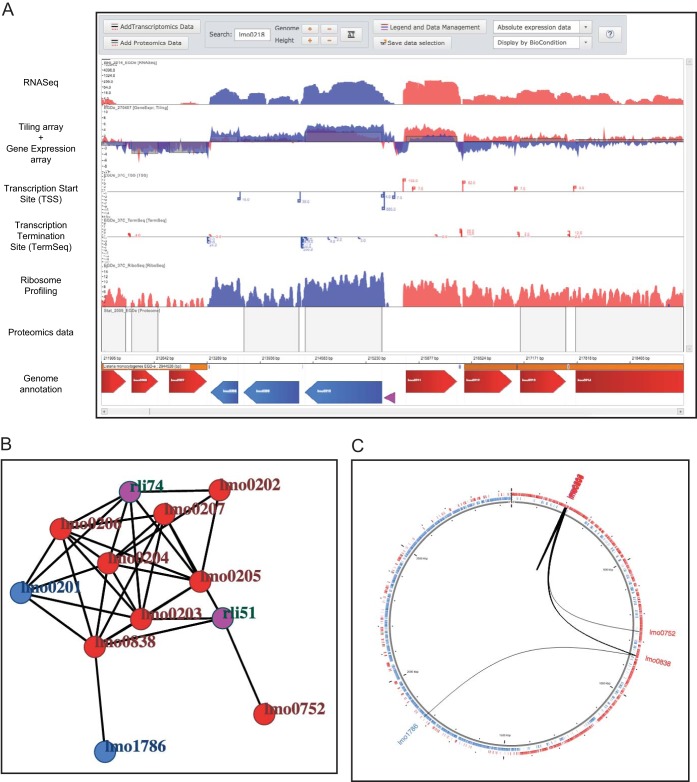
Multi-omics genome viewer and coexpression network tool. (A) Genome viewer of representative omics data sets for *L. monocytogenes* EGD-e grown in BHI at 37°C to the exponential and stationary growth phases as indicated in the text. The genome viewer shows positive genome strand genes (in red), negative genome strand genes (blue), tRNAs and rRNAs (in yellow), small RNAs (in purple), riboswitches (in green), asRNAs (in light green), predicted operons (in orange) from reference 56, and predicted transcription terminators (22) (in blue circles). Exp, exponential. (B) Coexpression network of the virulence locus genes (*lmo0200* to *lmo0207*) of *L. monocytogenes* EGD-e. Network nodes are genome elements (genes and noncoding RNAs) with the same color code as in the genome viewer tool. (C) Circular graph visualization of the coexpression network of the virulence locus genes (*lmo0200* to *lmo0207*). Coexpression edges are displayed overlaid on a circular representation of the EGD-e genome.

The coexpression network interface is designed to display possible correlations between genes and small RNAs in accordance with the “guilty by association” paradigm (60). This paradigm states that two genomic features that share the same expression profile might be involved in the same functional process. Pearson correlation coefficients have been calculated for 42 tiling array and RNA-Seq data sets. Once a specific Pearson correlation coefficient cutoff has been selected, this interface filters the coexpression network to show only the selected genome elements and the genome elements having a correlation coefficient above the selected cutoff. Two types of displays are available for the coexpression network, including a standard force-directed graph visualization (Fig. 3B) and a circular graph visualization (Fig. 3C). The latter viewer shows the Pearson correlation coefficient between genome elements by using a representation of the circular bacterial genome. This visualization highlights possible coexpression between distant genomic loci.

The Listeriomics database contains 74 proteomics data sets (Fig. 2D). All these data can be accessed through a summary panel with a search interface. Users can visualize the selected data sets on a heat map showing the presence or absence of each *Listeria* protein under a specific biological condition. Contrary to transcriptomics data sets, relative protein expression values are not available.

A webpage form is available to inform of the need to integrate a specific data set in the Listeriomics database. Before the publication of new genomes, transcriptomes, or proteomes to the Listeriomics website, the data sets must first be uploaded on referent repositories such as the Sequence Read Archive (61) or ArrayExpress (21). This process ensures that all the data sets are formatted in accordance with international standards and that minimal information on the experimental design of each study has been provided.

### The multi-omics genome viewer.

One of the key features of the Listeriomics interface is the multi-omics genome viewer. Figure 3A shows a variety of omics data sets produced for *L. monocytogenes* EGD-e grown at 37°C to the exponential phase in brain heart infusion (BHI) medium. RNA-Seq (62), tiling array (56), gene expression array (56), TSS (22), transcription termination site and ribosome profiling (63), and proteomics (36) data sets are displayed. To our knowledge, this is the first time that such a variety of omics scales can be browsed together through a genome viewer for a prokaryotic organism. Thus, the user can visualize the correlation of the genome annotation with transcription and translation for a specific coding RNA or sRNA. The genome viewer is dynamic, with zoom-in and zoom-out capabilities. The viewer also has search capability to access a specific position in the selected *Listeria* strain genome. Every omics data set present in the Listeriomics database can be added to this genome viewer.

Easy-access buttons on the home page of the Listeriomics interface allow quick access to three genome viewers with preloaded reference omics data sets. The first genome viewer is for *L. monocytogenes* EGD-e grown to the exponential phase at 37°C in BHI (Fig. 3A), the second is for the same condition but in stationary phase, and the last is for *L. monocytogenes* EGD-e grown in mouse macrophage cells.

### Meta-analysis of *Listeria* transcriptomic data sets.

**FIG 4:**
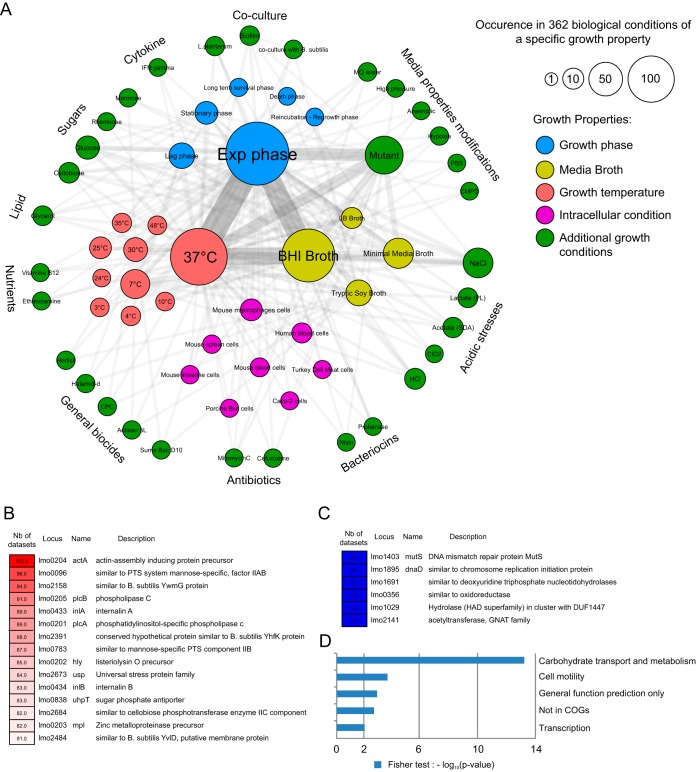
Meta-analysis of the Listeriomics transcriptomic data sets. (A) Relational network built on the 362 transcriptomic biological conditions found in the Listeriomics database. Each node corresponds to a growth condition. The size of each node is proportional to the occurrence of each condition in the whole database. A link is drawn between two growth conditions if they are present in the same transcriptomic data set. (B) Heat map of the 15 genes with the highest ratio of differential expression. The value used for colorization is the number of data sets in which each gene has been found to be differently expressed. (C) Heat map of the six genes with no variability. (D) Pathway enrichment analysis of the 651 genes of *L. monocytogenes* EGD-e that are found differently expressed in >10% of the 279 data sets. We performed a pathway enrichment analysis by using COG information and the Fisher exact test *P* value.

A meta-analysis of the diversity of transcriptomic data sets available in the Listeriomics database offers a striking overview of the variety of studies performed by the *Listeria* research community. This wide range of studies covers a majority of the different living environments in which *Listeria* species have been observed to grow. As shown in Fig. 4A, several studies on the effects of different growth environments on *Listeria* have been performed, i.e., cold environments, acidic environments, specific gene deletions, common biocides, sugar availability, and intracellular growth. This variety of biological conditions can be browsed easily on the Listeriomics website. Notably, the most frequently used growth condition is bacterial growth to the exponential phase at 37°C in BHI. This biological condition is used as a reference condition in most of the studies.

We extracted the list of genes of *L. monocytogenes* EGD-e found to be differently expressed in the highest number of data sets (Fig. 4B; see Table S6). Of the top 15 genes on this list, 7 are well-studied virulence genes (*actA*, *hly*, *plcA*, *plcB*, *mpl*, *inlA*, *inlB*, *uhpT*) all regulated by the PrfA protein. We identified *actA* as the most variable gene. This gene is differently expressed in 102 of the 279 available data sets for this *Listeria* strain. Among several other functions (64–66), the ActA protein is responsible for actin nucleation and intracellular propulsion. The other genes on the list are involved in either sugar metabolism (*lmo0096*, *lmo2391*, *lmo0783*, *lmo2684*) or stress response (*lmo2158*, *lmo2673*). Finally, a not-yet-described membrane protein, Lmo2484, is differently expressed in 81 of the 279 available data sets. This protein is conserved with >90% similarity in all 83 *Listeria* strains present in the Listeriomics database. We also found six genes that have never been demonstrated to be differently expressed in a data set (Fig. 4C; see Table S6). As expected, these genes are involved in general bacterial physiology factors like DNA mismatch repair or reductase and transferase enzymes.

Finally, we extracted the 651 genes of *L. monocytogenes* EGD-e that were found to be differently expressed in >10% of the 279 data sets (see Table S6). We performed a pathway enrichment analysis by using COG (Clusters of Orthologous Groups) information and the Fisher exact test (67) (Fig. 4D). We found that the cluster of genes differently expressed in the highest number of data sets was associated with the carbohydrate transport and metabolism functions (*P* = 4.15e-14). This may be linked to the fact that, to survive in a wide variety of environments, *Listeria* bacteria have to be able to switch from one carbon source to another. The second most represented cluster is that of cell motility genes (*P* = 2.18e-4). The third (*P* = 1.3e-3) and fourth (*P* = 2.1e-3) most represented clusters include genes without any specific function, most of them being annotated as encoding hypothetical proteins. The latter result highlights the fact that many of the important genes of *Listeria* species gene regulatory networks remain to be described. The Listeriomics resource is an essential tool for their investigation.

### Meta-analysis of *Listeria* proteomic data sets.

An analysis of the variety of proteomic data sets available in the Listeriomics database shows that many reference biological conditions have been studied. As for transcriptomic data, there is a great number of data sets produced by bacteria grown to the exponential phase at 37°C in BHI. Users can also access data sets from intracellular growth, cold environments, and the stationary growth phase. The key information in these proteomic data sets is the protein extraction protocol used that indicates which compartment of the bacterial cells (cytoplasm, membrane, cell wall, and secretome) has been analyzed. The cytoplasmic compartment is the most studied. The second most studied is the secretome compartment, the focus of many publications, since secreted proteins are key components of the *Listeria* intracellular life cycle. The ability to visualize these proteomic data sets together at the Listeriomics website will help the *Listeria* community to investigate further these groups of proteins and their roles in the different compartments of bacterial cells.

We investigated the 58 proteomic data sets available for *L. monocytogenes* EGD-e. We counted the data sets in which each protein was analyzed (see Table S6). As expected, we found that most of the proteins analyzed in half of the data sets come from the translation machinery or the carbohydrate transport and metabolism pathway. Remarkably, the first known virulence factor on the list is the pore-forming toxin LLO (Lmo0202), which is detected in 22 of the 58 proteomic data sets present in the Listeriomics database. We also found that >80% (2,344/2,859) of the *L. monocytogenes* EGD-e proteins have been observed in one or more proteomic data sets (see Table S6). Remarkably, 512 proteins were never shown to be produced.

10.1128/mSystems.00186-16.9 TEXT S1  References cited in the supplemental material. Download TEXT S1, DOCX file, 0.1 MB. Copyright © 2017 Bécavin et al. 2017 Bécavin et al. This content is distributed under the terms of the Creative Commons Attribution 4.0 International license.

### Systems-level analysis of the *L. monocytogenes* EGD-e virulence locus.

We investigated the coexpression network of the *L. monocytogenes* EGD-e virulence locus (*lmo0200* to *lmo0206*), linking every gene with a Pearson correlation coefficient of >0.85 (Fig. 3B, and C). As part of the PrfA core regulon (68), we found *uhpT* (*lmo0838*) and *inlC* (*lmo1786*) coexpressed with the virulence locus. Strikingly, the *prfA* gene (*lmo0200*) did not appear to be strictly coexpressed with the other genes of the virulence locus. Neither the *inlA* (*lmo0433*) nor the *inlB* (*lmo0444*) gene, both of which are extensively studied virulence factors regulated by the PrfA protein, was found in the coexpression network. The two noncoding RNA elements rli51 and rli74 in the virulence locus were also found to be coexpressed. In addition, we found another gene, *lmo0752*, coexpressed with the virulence locus that has not been described in the PrfA core regulon (68). Nevertheless, this gene was previously described (69) as part of the bile tolerance locus (*lmo0745* to *lmo0755*).

## DISCUSSION

The availability of curated information on genes, proteins, and cellular processes is essential not only for providing a better understanding of the *Listeria* genus but also as a key element in the development of our understanding of biological processes with respect to food industry and clinical applications. A systems biology approach requires the integration of a diversity of data collections, including, among others, genes, sRNAs, mRNAs, proteins, metabolites, protein-protein interactions, and protein-RNA interactions.

In the case of the *Listeria* genus, no such resource is currently available. At the species and strain levels, a first difficulty is indeed the recovery of these data sets in existing repositories, as is the case for *Listeria* proteomic data sets. In this way, the Listeriomics resource (http://listeriomics.pasteur.fr/) provides a unique, comprehensive, and up-to-date source of information on *Listeria*, including all the available complete genomes to date; a unified annotation of genes, proteins, and noncoding RNAs; and associated omics data and metadata relevant to genomic comparative and systems biology analyses. The curation process focused on the quality of the metadata provided. We completed the process by searching information in the corresponding publications. To our knowledge, the Listeriomics interface is the first bioinformatics resource designed to help scientists in *Listeria* pathogenesis research using a systems biology approach.

The information integrated into the Listeriomics database is provided through a useable and friendly interface that allows querying and visualization of *Listeria* genomic, phylogenetic, transcriptomic, and proteomic data sets, along with information on small RNAs and coexpression network of genes and small RNAs. The user experience and feedback from our collaborators using the Listeriomics interface for the last past 5 years were driving forces in organizing and improving the way to access data and tools (17, 22, 71). Indeed, through the Listeriomics resource, it is possible to summarize what has been discovered about a specific gene, including information on its distribution in the different *Listeria* genomes. Users can also identify the different transcriptomics data sets in which a specific gene or small RNA is differently expressed. Similarly, scientists can browse the different proteomics data sets to identify biological conditions in which a specific protein was detected by mass spectrometry. Finally, regulatory networks between genes and small RNAs can be inferred and explored.

The field of microbiology is considerably impacted by new technologies, and the role of databases in microbiology research will become even more important shortly (72). Until now, most of the model organism resources have focused on the widely studied bacterium *E. coli*, often with an emphasis on the manual curation of some particular aspect of omics information. Only a few of them, like the RegulonDB resource dedicated to the *E. coli* transcriptional regulatory network, really address the challenge of integrating knowledge based on experimental high-throughput omics data sets (50). For *B. subtilis*, SubtiWiki originally focused on manually curated gene annotation with a collection of pages providing interlinked pages on *B. subtilis* gene properties, including valuable information such as essentiality or sporulation, obtained from qualified members of the *Bacillus* community (73). Interestingly, the latest release of Subtiwiki integrates some omics data, with new modules allowing the linkage of pathway, interaction, and expression information (52). To our knowledge, none of these databases dedicated to model organisms such as *E. coli* or *B. subtilis* integrates as many data sets as the Listeriomics resource does. Moreover, Listeriomics is the only resource including such a variety of comparative and evolutionary analyses at the genomic level.

## MATERIALS AND METHODS

### Integration of *Listeria* species genomes and sRNAs.

The complete *Listeria* genomes available in the NCBI RefSeq and GenBank databases (see Table S1) were downloaded and integrated into our the Listeriomics database. Information about serotype, lineage, and the origin of strain isolation was included when possible. We used the PasteurMLST tool (74) to search for the sequence type and clonal complex of each strain. For *L. monocytogenes* EGD-e genes, we integrated their predicted functional group (11), COG, operon prediction (56), and subcellular protein localization prediction (27) (see Fig. S1).

10.1128/mSystems.00186-16.3 TABLE S1  List of *Listeria* genomes integrated into the website. A total of 83 complete *Listeria* genomes were downloaded from NCBI RefSeq and GenBank. All of the available chromosomes are listed with their names, sequence IDs, release dates, sizes, the numbers of coding elements, and the databases from which they were downloaded. Download TABLE S1, XLS file, 0.05 MB. Copyright © 2017 Bécavin et al. 2017 Bécavin et al. This content is distributed under the terms of the Creative Commons Attribution 4.0 International license.

Many studies have been performed to identify sRNAs in *L. monocytogenes* (22, 55–58). Among these studies, only one concerned strain 10403S (57); the others focused on EGD-e. Altogether, 304 noncoding RNA elements have now been reported in *L. monocytogenes*, of which 154 are sRNAs, 104 are asRNAs, and 46 are *cis-*regulatory elements (cisRegs) including riboswitches (see Table S2 and Fig. S1). We included these elements in our database, along with supplementary information gathered from all related publications. Prediction of secondary structures at 37°C for each noncoding RNA were calculated by using UNAFold software (hybrid-ss-min default parameters on the whole RNA [76]).

10.1128/mSystems.00186-16.4 TABLE S2  List of all 304 small RNAs discovered in *L. monocytogenes* EGD-e. Sheet 1 presents the set of 154 sRNAs, sheet 2 presents the set of 46 cisRegs, and sheet 3 presents the set of 104 asRNAs. For each set are shown the names and synonyms of the noncoding RNA elements, their positions, the first publication in which they were discovered, the others publications in which they were detected, and if they were detected in the TSS study. Download TABLE S2, XLS file, 0.1 MB. Copyright © 2017 Bécavin et al. 2017 Bécavin et al. This content is distributed under the terms of the Creative Commons Attribution 4.0 International license.

### *Listeria* ortholog gene families.

Nucleic and amino acid sequences of all the annotated coding genes were produced for each complete *Listeria* genome by using GenBank files and a custom-made Python script. Amino acid sequences for each *Listeria* genome were aligned against those of other proteins of all the *Listeria* genomes by using BLASTP+ (53) with an E-value threshold of 0.01. We used PanOCT (78) to build families of *Listeria* orthologs. PanOCT is able to deal with recently diverged paralogs by using neighborhood gene information. The percentage of genomes needed for a cluster to be considered an orthologous gene family was set to 100%. The length ratio to eliminate shorter protein fragments when a protein is split because of a frameshift event was set to 1.33 as previously recommended (78). Ortholog gene families were finally extracted from panoct.pl output files by using the gene_order.pl script, which is included in the PanOCT archive.

Amino acid sequences of each cluster were aligned by using ProbCons version 1.12 (79) with default parameters. Resulting alignments were postprocessed to filter unreliable positions by using Gblocks version 0.91b (80) with the parameter settings as follows. The minimum number of sequences for a conserved position was set to (*n*/2) + 1 (where *n* is the total number of sequences in the aligned data set), the maximum number of contiguous nonconserved positions was set to 50, the minimum length required for a block was set to 5, and gap positions were not allowed. The corresponding nucleic acid sequence alignments were obtained from each cluster amino acid sequence alignment with a custom-made Perl script.

### *Listeria* species reference phylogenomic tree.

Nucleic acid sequence alignments of ortholog gene families were concatenated into a single superalignment. This superalignment was used to compute a maximum-likelihood tree by using FastTree 2 (81), a parallelized and optimized software, to build maximum-likelihood trees. The following parameters were used. The generalized time-reversible model was chosen, the exhaustive search mode was selected to obtain a more accurate reconstruction, NNI and SPR heuristics were used to browse the tree space, and BIONJ weighting was chosen to join events during tree space browsing. Support analyses were performed with the Shimodaira-Hasegawa test associated with 1,000 resampling steps of site likelihood.

### Integration of transcriptomic data sets.

We downloaded the 64 published *Listeria-*related ArrayExpress experiments (21) in MAGE-TAB standard (82) (Fig. 1; see Table S3). Every MAGE-TAB file included an IDF (investigation description format) file showing general information about the experiment and an SDRF (sample and data relationship format) file showing data relationship. We manually curated every SDRF file and integrated the different metadata of the data set by using the publication linked to each study when available. We grouped the metadata information into key biological parameters for each data set: growth, time point, temperature, mutant, media, strain used, and strain array (Fig. 2A to C).

10.1128/mSystems.00186-16.5 TABLE S3  Summary of the transcriptomic data sets. Three excel spreadsheets are shown. The first one shows the 64 transcriptomic studies included in the Listeriomics database. For each study downloaded from ArrayExpress, the name, accession number, date, type of technology, *Listeria* strain used, and download URL are shown. On the second sheet is a list of the 38 available differential-expression data sets with related technologies (gene expression array, tiling array, RNA-Seq). The third sheet shows a summary of the RNA-Seq remapping performed with the percentage of covered reads, and the sequencing platform used. Download TABLE S3, XLS file, 0.1 MB. Copyright © 2017 Bécavin et al. 2017 Bécavin et al. This content is distributed under the terms of the Creative Commons Attribution 4.0 International license.

We then added matrices of expression or comparison for each biological condition. In total, 32 different gene expression arrays and six RNA-Seq technologies were combined. For gene expression arrays, we downloaded the 32 ADF (array design format) files that describe the relationship between array probes and genes. Only processed data provided by ArrayExpress were used, and no new normalization was applied. For each gene, we calculated the median differences of log values for relative expression tables. In the case of RNA-Seq, alignments of raw reads were performed with the Bowtie (83), segemehl (84), or novoalignCS tool, depending on the sequencing technology. For each alignment file, we computed the number of reads per kilobase per million mapped reads for each genomic feature and the number of reads per million for genome-wide coverage. Differential expression analysis was performed with DESeq version 1.14.0 (85) on per-feature raw counts.

Altogether 492 files, 150 absolute value data, and 342 relative expression data were created and integrated into the Listeriomics website (Fig. 1 and 2; see Fig. S1). For every experiment, relative expression data were always available, whereas absolute expression data were found for only a quarter of the data.

### Construction of an expression atlas.

We designed an expression atlas to provide for each transcriptomic data set a list of the differently expressed genome elements based on log fold change values. A statistical analysis (Shapiro-Wilk and Lilliefors tests) showed that all the relative expression data sets have a Gaussian distribution with a mean equal to zero. We applied normalization directly to log fold change values by multiplying every value by 1 divided by the square root of sigma, where sigma is the estimated standard deviation of each data set. This normalization ensured that every log fold change data set will have a standard deviation equal to 1 (Fig. S2). Consequently, the user can apply a standard cutoff, like log fold change < 1.5, and extract the differently expressed genome elements for every data set. A standard deviation equal to 1 leads, on average, to a selection of 7.1% of the most differently expressed genome elements, without removing the existing differences in the number of elements differently expressed under different biological conditions.

10.1128/mSystems.00186-16.2 FIG S2  Box plot before and after variance normalization of the transcriptomics data sets. (A) Box plot before variance normalization of the 255 relative expression (log fold change) transcriptomics data sets from *L. monocytogenes* EGD-e available in the Listeriomics database. (B) Box plot after variance normalization of the 255 relative expression (log fold change) transcriptomics data sets from *L. monocytogenes* EGD-e available in the Listeriomics database. A default log fold change cutoff of 1.5 will, after normalization, better discriminate the real differently expressed genome elements. Download FIG S2, EPS file, 1.5 MB. Copyright © 2017 Bécavin et al. 2017 Bécavin et al. This content is distributed under the terms of the Creative Commons Attribution 4.0 International license.

### Construction of coexpression networks.

We selected 42 transcriptomic data sets for which the absolute expression of genes and small RNAs was available (21 tiling array and 21 RNA-Seq [see Table S4 and Fig. S1]). The Pearson correlation coefficient for each genome element across all biological conditions was calculated. A link in the coexpression networks was created only when the absolute Pearson correlation coefficient was >0.80.

10.1128/mSystems.00186-16.6 TABLE S4  The 42 data sets used for reconstruction of the coexpression network of *L. monocytogenes* EGD-e. Displayed are the 42 transcriptomics data sets used for reconstruction of the coexpression network and all of the information about the biological conditions used. Download TABLE S4, XLS file, 0.03 MB. Copyright © 2017 Bécavin et al. 2017 Bécavin et al. This content is distributed under the terms of the Creative Commons Attribution 4.0 International license.

### Integration of proteomic data sets.

We selected 23 publications by using the PubMed database, in which a mass spectrometry experiment with a *Listeria* strain was performed (see Table S5). We extracted all the available information on the biological conditions screened in each experiment from associated articles and supplementary files (Fig. 1). We also extracted the metadata information in the same format for the transcriptomic data sets. In all the experiments, a list of the proteins detected was available. In total, we extracted 102 proteomics files (74 absolute expression data sets and 28 relative-expression data sets [Fig. 2D; see Fig. S1]).

10.1128/mSystems.00186-16.7 TABLE S5  The 23 proteomics data sets. Listed are the publications from which proteomics data were extracted. Publication dates, PubMed accession numbers, and download links are provided. Download TABLE S5, XLS file, 0.04 MB. Copyright © 2017 Bécavin et al. 2017 Bécavin et al. This content is distributed under the terms of the Creative Commons Attribution 4.0 International license.

10.1128/mSystems.00186-16.8 TABLE S6  Transcriptomic and proteomic metadata analysis of *L. monocytogenes* EGD-e genes. Three excel spreadsheets are shown. On the first, the number of transcriptomic data sets in which each gene is differently expressed is shown. On the second are the results of the pathway enrichment analysis performed on the 651 genes of *L. monocytogenes* EGD-e that are found differently expressed in >10% of the 279 data sets. We performed a pathway enrichment analysis by using COG information and a Fisher exact test. The third sheet displays the number of data sets in which each *L. monocytogenes* EGD-e protein has been detected. A histogram helps to summarize the distribution of protein detection. Download TABLE S6, XLS file, 1.5 MB. Copyright © 2017 Bécavin et al. 2017 Bécavin et al. This content is distributed under the terms of the Creative Commons Attribution 4.0 International license.

### Construction of the Listeriomics website and desktop versions.

The Listeriomics website was built by using the BACNET development platform (unpublished data). This platform is based on Java and Eclipse e4 RCP/RAP API for building both web and desktop software versions and provides a rich user interface. This open-source platform is generic and can be used to set up a similar website for any organism.

Tutorials for the Listeriomics website can be found on the Listeriomics mediaWiki webpage. These tutorials can be accessed either from the home webpage or directly at http://wiki.listeriomics.com/.
