# Supplementary material for: Listeriomics: an Interactive Web Platform for Systems Biology of Listeria
Source: mSystems. 2017 Mar 14;2(2):e00186-16. doi: 10.1128/mSystems.00186-16 (PMC5350546; doi:10.1128/mSystems.00186-16)
Supplement: TEXT S1 [file sys002172096s9.docx]

| **Reference in supplementary table** | **Table Number** | **Reference** |
| --- | --- | --- |
| Becavin et al., MBio 2014 (Pubmed:24667708) | Table S1 | (1) |
| Briers et al., J. Bacteriol. 2011 (PubMed:21685277) | Table S1 | (2) |
| Buchrieser et al., J. Bacteriol. 2011 (Pubmed:22072644) | Table S1 | (3) |
| Casey et al., Front. Microbiol. 2014 (Pubmed:24616718) | Table S1 | (4) |
| Centoram et al., Genome. Announ. 2015 (PubMed:25744995) | Table S1 | (5) |
| Chatterjee et al., Int. J. Med. Microb. 2006 (Pubmed:16527541) | Table S1 | (6) |
| Chen et al., J. Bacteriol. 2011 (Pubmed:21551300) | Table S1 | (7) |
| Chen et al., J. Bacteriol. 2011 (Pubmed:21742872) | Table S1 | (8) |
| den Bakker et al., Applied Env. Microb. 2012 (PubMed:22247147) | Table S1 | (9) |
| den Bakker., BMC Genomics 2010 (PubMed:21126366) | Table S1 | (10) |
| Fang et al., J. Micro. Biotech. 2016 (PubMed:26464378) | Table S1 | (11) |
| Gilmour et al., BMC Genomics 2010 (Pumed:20167121) | Table S1 | (12) |
| Glaser et al., Science 2001 (Pubmed:11679669) | Table S1 | (13) |
| Haase et al., Env. Microbiology 2011 (Pubmed:22003999) | Table S1 | (14) |
| Hain et al., BMC Genomics 2012 (Pubmed:22530965) | Table S1 | (15) |
| Hain et al., J. Bacteriol. 2006 (PubMed:16936040) | Table S1 | (16) |
| Holch et al., Appl. Env. Microb. 2013 (Pubmed:23435887) | Table S1 | (17) |
| Hupfeld et al., Genome Announc. 2015 (PubMed:25614561) | Table S1 | (18) |
| Klumpp et al., Genome Announc. 2014 (Pubmed:24786957) | Table S1 | (19) |
| Lomonaco et al., Emerging Infct. Dis. 2013 (PubMed:23260778) | Table S1 | (20) |
| Lyytikainen et al., J. Infect. Dis. 2000 (Pubmed:10823797) | Table S1 | (21) |
| Mc Mullen et al., J. Bacteriol. 2012 (Pumed:22689239) | Table S1 | (22) |
| Nelson et al., N.A.R. 2004 (Pubmed:15115801) | Table S1 | (23) |
| Olsen et al., Clin. Infect. Dis. 2005 (Pubmed:15824987) | Table S1 | (24) |
| Rupp et al., Vet. Microbio. 2015 (PubMed:25813546) | Table S1 | (25) |
| Schmitz-Esser et al., Genome Announc. 2015 (PubMed:25792065) | Table S1 | (26) |
| Steele et al., J. Bacteriol. 2011 (Pubmed:21602330) | Table S1 | (27) |
| Steinweg et al., J. Bacteriol. 2010 (PubMed:20061480) | Table S1 | (28) |
| Tan et al., Genome announc. 2015 (PubMed:25593254) | Table S1 | (29) |
| Tasara et al., Genome announc. 2014 (PubMed:25477407) | Table S1 | (30) |
| Tasara et al., Genome announc. 2015 (PubMed:26021930) | Table S1 | (31) |
| Tasara et al., Genome announc. 2016 (PubMed:26966206) | Table S1 | (32) |
| Weinmaier et al., Genome Announc. 2013 (Pubmed:23405339) | Table S1 | (33) |
| Barry et al. 1999 (PubMed:10220880) | Table S2 | (34) |
| Christiansen et al. 2006 (PubMed:16682563) | Table S2 | (35) |
| Johansson et al. 2002 (PubMed:12230973) | Table S2 | (36) |
| Johansson et al. 2009 (PubMed:19914169) | Table S2 | (37) |
| Mandin et al. 2007 (PubMed:17259222) | Table S2 | (38) |
| Mraheil et al. 2011 (PubMed:21278422) | Table S2 | (39) |
| Nielsen et al. 2008 (PubMed:18621897) | Table S2 | (40) |
| Oliver et al. 2009 (PubMed:20042087) | Table S2 | (40) |
| Toledo-Arana et al. 2009 (PubMed:19448609) | Table S2 | (41) |
| Wurtzel et al. 2012 (PubMed:22617957) | Table S2 | (42) |
| KEGG DAS | Table S2 | (43) |
| Rfam 2012 | Table S2 | (44) |
| Mraheil et al., N.A.R. 2011 (Pubmed:21278422) | Table S3 | (39) |
| Wehner et al., Plos One 2014 (Pubmed:25286309) | Table S3 | (45) |
| Laursen et al., Env. Microb. 2014 (Pubmed:24920558) | Table S3 | (46) |
| Mellin et al., Science 2014 (Pubmed:25146292) | Table S3 | (47) |
| Salazar et al., Appl. Env. Microb. 2013 (Pubmed:23835178) | Table S3 | (48) |
| Oliver et al., BMC Genomics 2009 (Pubmed:20042087) | Table S3 | (40) |
| Wehner et al., Plos One 2014 (Pubmed:25286309) | Table S4 | (45) |
| Toledo-arana et al., Nature 2009 (Pubmed:19448609) | Table S4 | (41) |
| Becavin et al., MBio 2014 (Pubmed:24667708) | Table S4 | (1) |
| Wurtzel et al., MSB 2012 (Pubmed:22617957) | Table S4 | (42) |
| Laursen et al., Env. Microb. 2014 (Pubmed:24920558) | Table S4 | (46) |
| Mellin et al., Science 2014 (Pubmed:25146292) | Table S4 | (47) |
| Salazar et al., Appl. Env. Microb. 2013 (Pubmed:23835178) | Table S4 | (48) |
| Behrens et al., Plos One 2014 (Pubmed:24498259) | Table S4 | (49) |
| ArrayExpress | Table S3 | (50) |
| Folio et al., Proteomics 2004 | Table S4 | (51) |
| Calvo et al., Proteomics 2004 | Table S4 | (52) |
| Trost et al., Proteomics 2004 | Table S4 | (53) |
| Wehmhoner et al., Electrohporesis 2005 | Table S4 | (54) |
| Dietrich et al., NAR 2005 | Table S4 | (55) |
| Abram et al., Appl. Env. Microb. 2008 | Table S4 | (56) |
| Dumas et al., Appl. Env. Microb. 2008 | Table S4 | (57) |
| Donaldson et al., Appl. Env. Microb. 2009 | Table S4 | (58) |
| Agoston et al., FoddBorne Path. And Dis. 2009 | Table S4 | (59) |
| Van de Velde et al., Proteomics 2009 | Table S4 | (60) |
| Cacace et al., J. Proteomics 2010 | Table S4 | (61) |
| Desvaux et al., J. Proteome Res. | Table S4 | (62) |
| Porteus et al., Appl. Micro. Biotech. 2011 | Table S4 | (63) |
| Donaldson et al., J. Proteomics 2011 | Table S4 | (64) |
| Garcia-del Portillo et al., J. Bio. Chemistry 2011 | Table S4 | (65) |
| Zhou et al., Curr. Microb. 2012 | Table S4 | (66) |
| Renier et al., Plos One 2012 | Table S4 | (67) |
| Melo et al., Int. J. Food. Microbiol. 2013 | Table S4 | (68) |
| Renier et al., J. Proteomics 2013 | Table S4 | (69) |
| Quereda et al., Microbiology 2013 | Table S4 | (70) |
| Zhang et al., Proteomics 2013 | Table S4 | (71) |
| Lee et al., Plos One 2013 | Table S4 | (72) |
| Pittman et al., J. Proteome 2014 | Table S4 | (73) |
| Halbedel et al., Mol. Cell. Proteomics 2014 | Table S4 | (74) |
| Mata et al., J. Proteome Res. 2015 | Table S4 | (75) |
| Tiong et al., J. Microbiological Meth. 2015 | Table S4 | (76) |

1. **Bécavin C**, **Bouchier C**, **Lechat P**, **Archambaud C**, **Creno S**, **Gouin E**, **Wu Z**, **Kühbacher A**, **Brisse S**, **Pucciarelli MG**, **García-del Portillo F**, **Hain T**, **Portnoy DA**, **Chakraborty T**, **Lecuit M**, **Pizarro-Cerdá J**, **Moszer I**, **Bierne H**, **Cossart P**. 2014. Comparison of widely used Listeria monocytogenes strains EGD, 10403S, and EGD-e highlights genomic variations underlying differences in pathogenicity. MBio **5**:e00969-14.

2. **Briers Y**, **Klumpp J**, **Schuppler M**, **Loessner MJ**. 2011. Genome sequence of Listeria monocytogenes Scott A, a clinical isolate from a food-borne listeriosis outbreak. J Bacteriol **193**:4284–5.

3. **Buchrieser C**, **Rusniok C**, **Garrido P**, **Hain T**, **Scortti M**, **Lampidis R**, **Karst U**, **Chakraborty T**, **Cossart P**, **Kreft J**, **Vazquez-Boland JA**, **Goebel W**, **Glaser P**. 2011. Complete Genome Sequence of the Animal Pathogen Listeria ivanovii, Which Provides Insights into Host Specificities and Evolution of the Genus Listeria. J Bacteriol **193**:6787–6788.

4. **Casey A**, **Fox EM**, **Schmitz-Esser S**, **Coffey A**, **McAuliffe O**, **Jordan K**. 2014. Transcriptome analysis of Listeria monocytogenes exposed to biocide stress reveals a multi-system response involving cell wall synthesis, sugar uptake, and motility. Front Microbiol **5**.

5. **Centorame P**, **Acciari VA**, **Orsini M**, **Torresi M**, **Iannetti L**, **Angius A**, **Di Giammartino D**, **Prencipe VA**, **Migliorati G**. 2015. Whole-Genome Sequence of Listeria monocytogenes Serovar 4b Strain IZSAM_Lm_hs2008, Isolated from a Human Infection in Italy. Genome Announc **3**:e00053-15.

6. **Chatterjee SS**, **Otten S**, **Hain T**, **Lingnau A**, **Carl UD**, **Wehland J**, **Domann E**, **Chakraborty T**. 2006. Invasiveness is a variable and heterogeneous phenotype in Listeria monocytogenes serotype strains. Int J Med Microbiol **296**:277–86.

7. **Chen Y**, **Strain E a**, **Allard M**, **Brown EW**. 2011. Genome sequences of Listeria monocytogenes strains J1816 and J1-220 associated with human outbreaks. J Bacteriol.

8. **Chen J**, **Xia Y**, **Cheng C**, **Fang C**, **Shan Y**, **Jin G**, **Fang W**. 2011. Genome sequence of the nonpathogenic Listeria monocytogenes serovar 4a strain M7. J Bacteriol **193**:5019–20.

9. **den Bakker HC**, **Bowen BM**, **Rodriguez-Rivera LD**, **Wiedmann M**. 2012. FSL J1-208, a virulent uncommon phylogenetic lineage IV Listeria monocytogenes strain with a small chromosome size and a putative virulence plasmid carrying internalin-like genes. Appl Environ Microbiol **78**:1876–89.

10. **den Bakker HC**, **Cummings CA**, **Ferreira V**, **Vatta P**, **Orsi RH**, **Degoricija L**, **Barker M**, **Petrauskene O**, **Furtado MR**, **Wiedmann M**. 2010. Comparative genomics of the bacterial genus Listeria: Genome evolution is characterized by limited gene acquisition and limited gene loss. BMC Genomics **11**:688.

11. **Fang C**, **Cao T**, **Shan Y**, **Xia Y**, **Xin Y**, **Cheng C**, **Song H**, **Bowman J**, **Li X**, **Zhou X**, **Fang W**. 2016. Comparative Genomic Analysis Reveals That the 20K and 38K Prophages in Listeria monocytogenes Serovar 4a Strains Lm850658 and M7 Contribute to Genetic Diversity but Not to Virulence. J Microbiol Biotechnol **26**:197–206.

12. **Gilmour MW**, **Graham M**, **Van Domselaar G**, **Tyler S**, **Kent H**, **Trout-Yakel KM**, **Larios O**, **Allen V**, **Lee B**, **Nadon C**. 2010. High-throughput genome sequencing of two Listeria monocytogenes clinical isolates during a large foodborne outbreak. BMC Genomics **11**:120.

13. **Glaser P**, **Frangeul L**, **Buchrieser C**, **Rusniok C**, **Amend A**, **Baquero F**, **Berche P**, **Bloecker H**, **Brandt P**, **Chakraborty T**, **Charbit A**, **Chetouani F**, **Couvé E**, **de Daruvar A**, **Dehoux P**, **Domann E**, **Domínguez-Bernal G**, **Duchaud E**, **Durant L**, **Dussurget O**, **Entian KD**, **Fsihi H**, **García-del Portillo F**, **Garrido P**, **Gautier L**, **Goebel W**, **Gómez-López N**, **Hain T**, **Hauf J**, **Jackson D**, **Jones LM**, **Kaerst U**, **Kreft J**, **Kuhn M**, **Kunst F**, **Kurapkat G**, **Madueno E**, **Maitournam A**, **Vicente JM**, **Ng E**, **Nedjari H**, **Nordsiek G**, **Novella S**, **de Pablos B**, **Pérez-Diaz JC**, **Purcell R**, **Remmel B**, **Rose M**, **Schlueter T**, **Simoes N**, **Tierrez A**, **Vázquez-Boland J a**, **Voss H**, **Wehland J**, **Cossart P**. 2001. Comparative genomics of Listeria species. Science (80- ) **294**:849–52.

14. **Haase JK**, **Murphy R a**, **Choudhury KR**, **Achtman M**. 2011. Revival of Seeliger’s historical “Special Listeria Culture Collection”. Environ Microbiol **13**:3163–71.

15. **Hain T**, **Ghai R**, **Billion A**, **Kuenne CT**, **Steinweg C**, **Izar B**, **Mohamed W**, **Mraheil MA**, **Domann E**, **Schaffrath S**, **Kärst U**, **Goesmann A**, **Oehm S**, **Pühler A**, **Merkl R**, **Vorwerk S**, **Glaser P**, **Garrido P**, **Rusniok C**, **Buchrieser C**, **Goebel W**, **Chakraborty T**. 2012. Comparative genomics and transcriptomics of lineages I, II, and III strains of Listeria monocytogenes. BMC Genomics **13**:144.

16. **Hain T**, **Steinweg C**, **Kuenne CT**, **Billion A**, **Ghai R**, **Chatterjee SS**, **Domann E**, **Kärst U**, **Goesmann A**, **Bekel T**, **Bartels D**, **Kaiser O**, **Meyer F**, **Pühler A**, **Weisshaar B**, **Wehland J**, **Liang C**, **Dandekar T**, **Lampidis R**, **Kreft J**, **Goebel W**, **Chakraborty T**. 2006. Whole-genome sequence of Listeria welshimeri reveals common steps in genome reduction with Listeria innocua as compared to Listeria monocytogenes. J Bacteriol **188**:7405–15.

17. **Holch A**, **Webb K**, **Lukjancenko O**, **Ussery D**, **Rosenthal BM**, **Gram L**. 2013. Genome sequencing identifies two nearly unchanged strains of persistent Listeria monocytogenes isolated at two different fish processing plants sampled 6 years apart. Appl Environ Microbiol **79**:2944–51.

18. **Hupfeld M**, **Fouts DE**, **Loessner MJ**, **Klumpp J**. 2015. Genome Sequences of the Listeria ivanovii subsp. ivanovii Type Strain and Two Listeria ivanovii subsp. londoniensis Strains. Genome Announc **3**:e01440-14.

19. **Klumpp J**, **Staubli T**, **Schmitter S**, **Hupfeld M**, **Fouts DE**, **Loessner J**. 2014. Genome Sequences of Three Frequently Used Listeria monocytogenes and Listeria ivanovii Strains **2**:4–5.

20. **Lomonaco S**, **Verghese B**, **Gerner-Smidt P**, **Tarr C**, **Gladney L**, **Joseph L**, **Katz L**, **Turnsek M**, **Frace M**, **Chen Y**, **Brown E**, **Meinersmann R**, **Berrang M**, **Knabel S**. 2013. Novel epidemic clones of Listeria monocytogenes, United States, 2011. Emerg Infect Dis **19**:147–50.

21. **Lyytikäinen O**, **Autio T**, **Maijala R**, **Ruutu P**, **Honkanen-Buzalski T**, **Miettinen M**, **Hatakka M**, **Mikkola J**, **Anttila VJ**, **Johansson T**, **Rantala L**, **Aalto T**, **Korkeala H**, **Siitonen A**. 2000. An outbreak of Listeria monocytogenes serotype 3a infections from butter in Finland. J Infect Dis **181**:1838–41.

22. **McMullen PD**, **Gillaspy AF**, **Gipson J**, **Bobo LD**, **Skiest DJ**, **Freitag NE**. 2012. Genome sequence of Listeria monocytogenes 07PF0776, a cardiotropic serovar 4b strain. J Bacteriol **194**:3552.

23. **Nelson KE**, **Fouts DE**, **Mongodin EF**, **Ravel J**, **DeBoy RT**, **Kolonay JF**, **Rasko DA**, **Angiuoli S V**, **Gill SR**, **Paulsen IT**, **Peterson J**, **White O**, **Nelson WC**, **Nierman W**, **Beanan MJ**, **Brinkac LM**, **Daugherty SC**, **Dodson RJ**, **Durkin AS**, **Madupu R**, **Haft DH**, **Selengut J**, **Van Aken S**, **Khouri H**, **Fedorova N**, **Forberger H**, **Tran B**, **Kathariou S**, **Wonderling LD**, **Uhlich GA**, **Bayles DO**, **Luchansky JB**, **Fraser CM**. 2004. Whole genome comparisons of serotype 4b and 1/2a strains of the food-borne pathogen Listeria monocytogenes reveal new insights into the core genome components of this species. Nucleic Acids Res **32**:2386–95.

24. **Olsen SJ**, **Patrick M**, **Hunter SB**, **Reddy V**, **Kornstein L**, **MacKenzie WR**, **Lane K**, **Bidol S**, **Stoltman GA**, **Frye DM**, **Lee I**, **Hurd S**, **Jones TF**, **LaPorte TN**, **Dewitt W**, **Graves L**, **Wiedmann M**, **Schoonmaker-Bopp DJ**, **Huang AJ**, **Vincent C**, **Bugenhagen A**, **Corby J**, **Carloni ER**, **Holcomb ME**, **Woron RF**, **Zansky SM**, **Dowdle G**, **Smith F**, **Ahrabi-Fard S**, **Ong AR**, **Tucker N**, **Hynes NA**, **Mead P**. 2005. Multistate outbreak of Listeria monocytogenes infection linked to delicatessen turkey meat. Clin Infect Dis **40**:962–7.

25. **Rupp S**, **Aguilar-Bultet L**, **Jagannathan V**, **Guldimann C**, **Drögemüller C**, **Pfarrer C**, **Vidondo B**, **Seuberlich T**, **Frey J**, **Oevermann A**. 2015. A naturally occurring prfA truncation in a Listeria monocytogenes field strain contributes to reduced replication and cell-to-cell spread. Vet Microbiol **179**:91–101.

26. **Schmitz-Esser S**, **Gram L**, **Wagner M**. 2015. Complete Genome Sequence of the Persistent Listeria monocytogenes Strain R479a. Genome Announc **3**:e00150-15.

27. **Steele CL**, **Donaldson JR**, **Paul D**, **Banes MM**, **Arick T**, **Bridges SM**, **Lawrence ML**. 2011. Genome sequence of lineage III Listeria monocytogenes strain HCC23. J Bacteriol **193**:3679–80.

28. **Steinweg C**, **Kuenne CT**, **Billion A**, **Mraheil MA**, **Domann E**, **Ghai R**, **Barbuddhe SB**, **Kärst U**, **Goesmann A**, **Pühler A**, **Weisshaar B**, **Wehland J**, **Lampidis R**, **Kreft J**, **Goebel W**, **Chakraborty T**, **Hain T**. 2010. Complete genome sequence of Listeria seeligeri, a nonpathogenic member of the genus Listeria. J Bacteriol **192**:1473–4.

29. **Tan W**, **Wang G**, **Pan Z**, **Yin Y**, **Jiao X**. 2015. Complete Genome Sequence of Listeria monocytogenes NTSN, a Serovar 4b and Animal Source Strain. Genome Announc **3**:e01403-14.

30. **Tasara T**, **Weinmaier T**, **Klumpp J**, **Rattei T**, **Stephan R**. 2014. Complete Genome Sequence of Listeria monocytogenes Lm60, a Strain with an Enhanced Cold Adaptation Capacity. Genome Announc **2**:e01248-14-e01248-14.

31. **Tasara T**, **Ebner R**, **Klumpp J**, **Stephan R**. 2015. Complete Genome Sequence of Listeria monocytogenes N2306, a Strain Associated with the 2013-2014 Listeriosis Outbreak in Switzerland. Genome Announc **3**:e00553-15.

32. **Tasara T**, **Klumpp J**, **Bille J**, **Stephan R**. 2016. Genome Sequences of Listeria monocytogenes Strains Responsible for Cheese- and Cooked Ham Product-Associated Swiss Listeriosis Outbreaks in 2005 and 2011. Genome Announc **4**:e00106-16.

33. **Weinmaier T**, **Riesing M**, **Rattei T**, **Bille J**, **Arguedas-Villa C**, **Stephan R**, **Tasara T**. 2013. Complete Genome Sequence of Listeria monocytogenes LL195, a Serotype 4b Strain from the 1983-1987 Listeriosis Epidemic in Switzerland. Genome Announc **1**:e00152-12-e00152-12.

34. **Barry T**, **Kelly M**, **Glynn B**, **Peden J**. 1999. Molecular cloning and phylogenetic analysis of the small cytoplasmic RNA from Listeria monocytogenes. FEMS Microbiol Lett **173**:47–53.

35. **Christiansen JK**, **Nielsen JS**, **Ebersbach T**, **Valentin-hansen P**, **Søgaard-andersen L**, **Kallipolitis BH**. 2006. Identification of small Hfq-binding RNAs in Listeria monocytogenes Identification of small Hfq-binding RNAs in Listeria monocytogenes. Rna **12**:1383–96.

36. **Johansson J**, **Mandin P**, **Renzoni A**, **Chiaruttini C**, **Springer M**, **Cossart P**. 2002. An RNA thermosensor controls expression of virulence genes in Listeria monocytogenes. Cell **110**:551–61.

37. **Loh E**, **Dussurget O**, **Gripenland J**, **Vaitkevicius K**, **Tiensuu T**, **Mandin P**, **Repoila F**, **Buchrieser C**, **Cossart P**, **Johansson J**. 2009. A trans-acting riboswitch controls expression of the virulence regulator PrfA in Listeria monocytogenes. Cell **139**:770–9.

38. **Mandin P**, **Repoila F**, **Vergassola M**, **Geissmann T**, **Cossart P**. 2007. Identification of new noncoding RNAs in Listeria monocytogenes and prediction of mRNA targets. Nucleic Acids Res **35**:962–74.

39. **Mraheil MA**. 2011. The intracellular sRNA transcriptome of Listeria monocytogenes during growth in macrophages. (supp table). Nucleic Acids Res 1323–1338.

40. **Oliver HF**, **Orsi RH**, **Ponnala L**, **Keich U**, **Wang W**, **Sun Q**, **Cartinhour SW**, **Filiatrault MJ**, **Wiedmann M**, **Boor KJ**. 2009. Deep RNA sequencing of L. monocytogenes reveals overlapping and extensive stationary phase and sigma B-dependent transcriptomes, including multiple highly transcribed noncoding RNAs. BMC Genomics **10**:641.

41. **Toledo-Arana A**, **Dussurget O**, **Nikitas G**, **Sesto N**, **Guet-Revillet H**, **Balestrino D**, **Loh E**, **Gripenland J**, **Tiensuu T**, **Vaitkevicius K**, **Barthelemy M**, **Vergassola M**, **Nahori M-A**, **Soubigou G**, **Régnault B**, **Coppée J-Y**, **Lecuit M**, **Johansson J**, **Cossart P**. 2009. The Listeria transcriptional landscape from saprophytism to virulence. Nature **459**:950–6.

42. **Wurtzel O**, **Sesto N**, **Mellin JR**, **Karunker I**, **Edelheit S**, **Bécavin C**, **Archambaud C**, **Cossart P**, **Sorek R**. 2012. Comparative transcriptomics of pathogenic and non-pathogenic Listeria species. Mol Syst Biol **8**:1–14.

43. **Kanehisa M**, **Furumichi M**, **Tanabe M**, **Sato Y**, **Morishima K**. 2017. KEGG: new perspectives on genomes, pathways, diseases and drugs. Nucleic Acids Res **45**:D353–D361.

44. **Daub J**, **Eberhardt RY**, **Tate JG**, **Burge SW**. 2015. Rfam: Annotating Families of Non-Coding RNA Sequences, p. 349–363. *In* Methods in molecular biology (Clifton, N.J.).

45. **Wehner S**, **Mannala GK**, **Qing X**, **Madhugiri R**, **Chakraborty T**, **Mraheil M a**, **Hain T**, **Marz M**. 2014. Detection of Very Long Antisense Transcripts by Whole Transcriptome RNA-Seq Analysis of Listeria monocytogenes by Semiconductor Sequencing Technology. PLoS One **9**:e108639.

46. **Laursen MF**, **Bahl MI**, **Licht TR**, **Gram L**, **Knudsen GM**. 2014. A single exposure to a sublethal pediocin concentration initiates a resistance-associated temporal cell envelope and general stress response in Listeria monocytogenes. Environ Microbiol.

47. **Mellin JR**, **Koutero M**, **Dar D**, **Nahori M -a.**, **Sorek R**, **Cossart P**. 2014. Sequestration of a two-component response regulator by a riboswitch-regulated noncoding RNA. Science (80- ) **345**:940–943.

48. **Salazar JK**, **Wu Z**, **McMullen PD**, **Luo Q**, **Freitag NE**, **Tortorello M Lou**, **Hu S**, **Zhang W**. 2013. PrfA-like transcription factor gene lmo0753 contributes to L-rhamnose utilization in Listeria monocytogenes strains associated with human food-borne infections. Appl Environ Microbiol **79**:5584–92.

49. **Behrens S**, **Widder S**, **Mannala GK**, **Qing X**, **Madhugiri R**, **Kefer N**, **Mraheil MA**, **Rattei T**, **Hain T**. 2014. Ultra Deep Sequencing of Listeria monocytogenes sRNA Transcriptome Revealed New Antisense RNAs. PLoS One **9**:e83979.

50. **Rustici G**, **Kolesnikov N**, **Brandizi M**, **Burdett T**, **Dylag M**, **Emam I**, **Farne A**, **Hastings E**, **Ison J**, **Keays M**, **Kurbatova N**, **Malone J**, **Mani R**, **Mupo A**, **Pedro Pereira R**, **Pilicheva E**, **Rung J**, **Sharma A**, **Tang YA**, **Ternent T**, **Tikhonov A**, **Welter D**, **Williams E**, **Brazma A**, **Parkinson H**, **Sarkans U**. 2013. ArrayExpress update--trends in database growth and links to data analysis tools. Nucleic Acids Res **41**:D987-90.

51. **Folio P**, **Chavant P**, **Chafsey I**, **Belkorchia A**, **Chambon C**, **Hébraud M**. 2004. Two-dimensional electrophoresis database of Listeria monocytogenes EGDe proteome and proteomic analysis of mid-log and stationary growth phase cells. Proteomics **4**:3187–201.

52. **Calvo E**, **Pucciarelli MG**, **Bierne H**, **Cossart P**, **Albar JP**, **García-Del Portillo F**. 2005. Analysis of the Listeria cell wall proteome by two-dimensional nanoliquid chromatography coupled to mass spectrometry. Proteomics **5**:433–43.

53. **Trost M**, **Wehmhöner D**, **Kärst U**, **Dieterich G**, **Wehland J**, **Jänsch L**. 2005. Comparative proteome analysis of secretory proteins from pathogenic and nonpathogenic Listeria species. Proteomics **5**:1544–57.

54. **Wehmhöner D**, **Dieterich G**, **Fischer E**, **Baumgärtner M**, **Wehland J**, **Jänsch L**. 2005. &quot;LaneSpector&quot;, a tool for membrane proteome profiling based on sodium dodecyl sulfate-polyacrylamide gel electrophoresis/liquid chromatography-tandem mass spectrometry analysis: application to Listeria monocytogenes membrane proteins. Electrophoresis **26**:2450–60.

55. **Dieterich G**, **Kärst U**, **Fischer E**, **Wehland J**, **Jänsch L**. 2006. LEGER: knowledge database and visualization tool for comparative genomics of pathogenic and non-pathogenic Listeria species. Nucleic Acids Res **34**:D402-6.

56. **Abram F**, **Su W-L**, **Wiedmann M**, **Boor KJ**, **Coote P**, **Botting C**, **Karatzas KAG**, **O’Byrne CP**. 2008. Proteomic analyses of a Listeria monocytogenes mutant lacking sigmaB identify new components of the sigmaB regulon and highlight a role for sigmaB in the utilization of glycerol. Appl Environ Microbiol **74**:594–604.

57. **Dumas E**, **Meunier B**, **Berdagué J-L**, **Chambon C**, **Desvaux M**, **Hébraud M**. 2008. Comparative analysis of extracellular and intracellular proteomes of Listeria monocytogenes strains reveals a correlation between protein expression and serovar. Appl Environ Microbiol **74**:7399–409.

58. **Donaldson JR**, **Nanduri B**, **Burgess SC**, **Lawrence ML**. 2009. Comparative proteomic analysis of Listeria monocytogenes strains F2365 and EGD. Appl Environ Microbiol **75**:366–73.

59. **Agoston R**, **Soni K**, **Jesudhasan PR**, **Russell WK**, **Mohácsi-Farkas C**, **Pillai SD**. 2009. Differential expression of proteins in Listeria monocytogenes under thermotolerance-inducing, heat shock, and prolonged heat shock conditions. Foodborne Pathog Dis **6**:1133–40.

60. **Van de Velde S**, **Delaive E**, **Dieu M**, **Carryn S**, **Van Bambeke F**, **Devreese B**, **Raes M**, **Tulkens PM**. 2009. Isolation and 2-D-DIGE proteomic analysis of intracellular and extracellular forms of Listeria monocytogenes. Proteomics **9**:5484–96.

61. **Cacace G**, **Mazzeo MF**, **Sorrentino A**, **Spada V**, **Malorni A**, **Siciliano R a**. 2010. Proteomics for the elucidation of cold adaptation mechanisms in Listeria monocytogenes. J Proteomics **73**:2021–30.

62. **Desvaux M**, **Dumas E**, **Chafsey I**, **Chambon C**, **Hébraud M**. 2010. Comprehensive Appraisal of the Extracellular Proteins from a Monoderm Bacterium: Theoretical and Empirical Exoproteomes of Listeria monocytogenes EGD-e by Secretomics.

63. **Porteus B**, **Kocharunchitt C**, **Nilsson RE**, **Ross T**, **Bowman JP**. 2011. Utility of gel-free, label-free shotgun proteomics approaches to investigate microorganisms. Appl Microbiol Biotechnol **90**:407–16.

64. **Donaldson JR**, **Nanduri B**, **Pittman JR**, **Givaruangsawat S**, **Burgess SC**, **Lawrence ML**. 2011. Proteomic expression profiles of virulent and avirulent strains of Listeria monocytogenes isolated from macrophages. J Proteomics **74**:1906–17.

65. **García-del Portillo F**, **Calvo E**, **D’Orazio V**, **Pucciarelli MG**. 2011. Association of ActA to peptidoglycan revealed by cell wall proteomics of intracellular Listeria monocytogenes. J Biol Chem **286**:34675–89.

66. **Zhou Q**, **Feng X**, **Zhang Q**, **Feng F**, **Yin X**, **Shang J**, **Qu H**, **Luo Q**. 2012. Carbon catabolite control is important for Listeria monocytogenes biofilm formation in response to nutrient availability. Curr Microbiol **65**:35–43.

67. **Renier S**, **Micheau P**, **Talon R**, **Hébraud M**, **Desvaux M**. 2012. Subcellular localization of extracytoplasmic proteins in monoderm bacteria: rational secretomics-based strategy for genomic and proteomic analyses. PLoS One **7**:e42982.

68. **Melo J**, **Schrama D**, **Hussey S**, **Andrew PW**, **Faleiro ML**. 2013. Listeria monocytogenes dairy isolates show a different proteome response to sequential exposure to gastric and intestinal fluids. Int J Food Microbiol **163**:51–63.

69. **Renier S**, **Chambon C**, **Viala D**, **Chagnot C**, **Hébraud M**, **Desvaux M**. 2013. Exoproteomic analysis of the SecA2-dependent secretion in Listeria monocytogenes EGD-e. J Proteomics **80**:183–195.

70. **Quereda JJ**, **Pucciarelli MG**, **Botello-Morte L**, **Calvo E**, **Carvalho F**, **Bouchier C**, **Vieira A**, **Mariscotti JF**, **Chakraborty T**, **Cossart P**, **Hain T**, **Cabanes D**, **García-Del Portillo F**. 2013. Occurrence of mutations impairing sigma factor B (SigB) function upon inactivation of Listeria monocytogenes genes encoding surface proteins. Microbiology mic.0.067744-0-.

71. **Zhang CXY**, **Creskey MC**, **Cyr TD**, **Brooks B**, **Huang H**, **Pagotto F**, **Lin M**. 2013. Proteomic identification of Listeria monocytogenes surface-associated proteins. Proteomics **13**:3040–5.

72. **Lee JH**, **Choi C-W**, **Lee T**, **Kim S Il**, **Lee J-C**, **Shin J-H**. 2013. Transcription factor σB plays an important role in the production of extracellular membrane-derived vesicles in Listeria monocytogenes. PLoS One **8**:e73196.

73. **Pittman JR**, **Buntyn JO**, **Posadas G**, **Nanduri B**, **Pendarvis K**, **Donaldson JR**. 2014. Proteomic Analysis of Cross Protection Provided between Cold and Osmotic Stress in Listeria monocytogenes.

74. **Halbedel S**, **Reiss S**, **Hahn B**, **Albrecht D**, **Mannala GK**, **Chakraborty T**, **Hain T**, **Engelmann S**, **Flieger A**. 2014. A systematic proteomic analysis of Listeria monocytogenes house-keeping protein secretion systems. Mol Cell Proteomics **13**:3063–81.

75. **Mata MM**, **da Silva WP**, **Wilson R**, **Lowe E**, **Bowman JP**. 2015. Attached and planktonic Listeria monocytogenes global proteomic responses and associated influence of strain genetics and temperature. J Proteome Res **14**:1161–73.

76. **Tiong HK**, **Hartson S**, **Muriana PM**. 2015. Comparison of five methods for direct extraction of surface proteins from Listeria monocytogenes for proteomic analysis by orbitrap mass spectrometry. J Microbiol Methods **110**:54–60.
